# Health of people with impairments and disabilities in Germany – Selected indicators from GEDA 2014/2015-EHIS

**DOI:** 10.25646/9752

**Published:** 2022-03-30

**Authors:** Franziska Prütz, Laura Krause

**Affiliations:** Robert Koch Institute, Berlin Department of Epidemiology and Health Monitoring

**Keywords:** DISABILITIES, IMPAIRMENTS, HEALTH BEHAVIOUR, HEALTH CARE, HEALTH MONITORING

## Abstract

A large part of the population is affected by impairments and disabilities. Around 13% of people in Germany have an officially recognised disability, and an estimated 15.6% have an impairment. This article provides an overview of the health of people with impairments and disabilities on the basis of selected indicators. The analyses are based on data from 23,372 participating persons aged 18 and over (12,747 women, 10,625 men) in the GEDA 2014/2015-EHIS study of the Robert Koch Institute (RKI), a nationwide survey of the adult population in Germany. 21.5% of persons with impairments and disabilities rate their health as good or very good, in contrast to 76.0% of persons without impairments and disabilities. Depressive symptoms exist in 27.1% of persons with impairments and disabilities and 7.5% of persons without impairments and disabilities. In part, there were differences in health behaviour, for example, people with impairments and disabilities do less aerobic physical activities and consume alcohol in risky amounts less often. 97.0% of the persons with and 86.1% of the persons without impairments and disabilities make use of outpatient medical services within one year, the former also have a higher inpatient and home care utilisation. Overall, poorer health is found among women than among men with impairments and disabilities, as well as with increasing age. The analyses show the need for prevention, health promotion and health care. Further data is needed to describe the health situation of people with impairments and disabilities.

## 1. Introduction

A large part of the population is affected by impairments and disabilities – directly or as relatives. In 2019, 10.4 million people with an officially recognised disability lived in private households in Germany, which corresponds to 12.7% of the residents living in private households [1]. 9.5% of people in Germany had an officially recognised severe disability, i.e. the degree of disability (GdB) was 50 or higher [2]. The proportion of people with impairments is much higher. It is estimated at about 15.6% of the population [3]. Definitions of disability, severe disability and impairment can be found in the Info box.

Article 25 of the United Nations Convention on the Rights of Persons with Disabilities (UNCRPD) stipulates ‘that persons with disabilities have the right to the enjoyment of the highest attainable standard of health without discrimination on the basis of disability’ [4]. Reliable data on the health of persons with disabilities is indispensable for identifying the need for political action. The UNCRPD also formulates corresponding requirements in Article 31 [4]. However, there is still only little data on the health of people with impairments and disabilities in Germany. The microcensus and the statistics on severely disabled persons provided by the Federal Statistical Office only include persons with officially recognised disabilities. The microcensus also only includes persons living in private households. The statistics on severely disabled persons provide information on the cause (e.g. accident or illness) and the type of disability (e.g. physical, visual, hearing, mental or learning disability), while the microcensus collects data on the social situation, for example on marital status, household size and educational qualifications, and also includes some questions on health every four years [5]. The Federal Government’s Reports on Participation [3, 6, 7] describe the health situation of people with impairments and disabilities, using data from the studies German Health Update (GEDA), German Health Interview and Examination Survey for Children and Adolescents (KiGGS), the Socio-Economic Panel (SOEP) and social security data. The reports Health in Germany [8] and Health Situation of Women in Germany [9] published by Federal Health Reporting, contain chapters on the health of people with impairments and disabilities, which are based on various data sources.

**GEDA 2014/2015-EHIS**

**Data holder:** Robert Koch Institute

**Aims:** To provide reliable information about the population’s health status, health-related behaviour and health care in Germany, with the possibility of a European comparison

**Method:** Questionnaires completed on paper or online

**Population:** People aged 18 years and above with permanent residency in Germany

**Sampling:** Registry office sample; randomly selected individuals from 301 communities in Germany were invited to participate

**Participants:** 24,016 people (13,144 women; 10,872 men)

**Response rate:** 26.9%

**Study period:** November 2014–July 2015

More information in German is available at www.geda-studie.de

People with impairments and disabilities are a heterogeneous group in which very different health situations and needs exist. Nevertheless, the existing data conveys the picture that they have poorer physical and mental health and thus have a higher need for health care, but at the same time there are gaps, for example with regard to the accessibility of medical practices [3, 9]. There are also differences in health behaviour between people with and without impairments and disabilities, partly in the direction of a more health-risky, partly in the direction of a more health-conscious behaviour [3, 9].

The aim of this article is to provide an overview of the health of people with impairments and disabilities on the basis of selected indicators. This includes indicators of health status, health behaviour as well as health care with data from the study GEDA 2014/2015-EHIS. The selection is based on the report Health Situation of Women in Germany [9], which was published in December 2020. Thus, the self-assessment of the general state of health contains important information for the description of the health of persons and population groups [8–10]. The presence of depressive symptoms gives an indication of mental health, as depression is one of the most common mental disorders [9, 11]. Aerobic physical activities (such as cycling, jogging or swimming), muscle-strengthening activities (such as strength training or yoga), fruit and vegetable consumption, smoking and risky alcohol consumption represent relevant aspects of health-related behaviour [9, 12]. The use of outpatient medical care, inpatient care and home care are reported as indicators of health care [9, 13, 14].

## 2. Methodology

### 2.1 Sample design and study implementation

The German Health Update (GEDA) is a nationwide survey of the adult population (aged 18 years and older) and part of the health monitoring program at the Robert Koch Institute (RKI). In GEDA 2014/2015, the questionnaire of the European Health Interview Survey (EHIS Wave 2) was fully integrated for the first time [15]. The survey was conducted by means of a self-completion questionnaire, which could be processed either as a paper or online version [16]. GEDA 2014/2015-EHIS is based on a two-stage stratified cluster sample. For this purpose, 301 municipalities were initially randomly selected. These account for 231 districts and district-free cities and represent the different municipality sizes and regions in Germany. In a second step, persons with permanent residence in the selected municipalities were randomly drawn from local population registers. Persons living in institutions or homes did not take part in the survey.

**Info box Persons with impairments and disabilities**

**1. Persons with impairments**

Persons with impairments are those who are permanently impaired in activities related to damage to body structures and functions. Depending on the data source, there are different statistical definitions for persons with impairments. Common to all of these groups, however, is that the persons belonging to them do not necessarily have to be restricted in their activities of everyday life due to their impairments, but they may nevertheless be so. [...]

**2. Persons with disabilities**

These are persons who are hindered in activities of daily living and/or equal participation by interactions of their own impairments and environmental barriers. It does not matter whether this is an officially recognised disability or severe disability. [...]

**3. Persons with recognised disability and recognised severe disability**

Persons with a recognised disability or a recognised severe disability include all persons whose disability has been determined or recognised by a competent office. Recognition is accompanied by the assignment of a degree of severity of disability in the form of a degree of disability (GdB). If a GdB of 50 or more has been assigned, this person has a recognised severe disability. [...]

Source: Federal Ministry of Labour and Social Affairs (2021) [3]

### 2.2 Indicators

For the analyses, the target variable on impairments and disabilities was operationalised as in the Second Report on Participation [7]: Persons with impairments and disabilities are understood to be all participants who have an officially recognised severe disability or a severe illness-related restriction in the performance of everyday activities lasting longer than six months. Participants were asked: ‘Do you have a disability that is officially recognised by the pension office?’ and, if the answer was ‘Yes’, ‘What is your officially recognised degree of disability?’ A GdB of 50 or more constitutes a severe disability. Impairments were recorded with the following question: ‘Are you permanently restricted by a health problem in activities of normal everyday life?’ If the answer to this question was ‘Yes’, the respondents were then asked about the severity (‘How severe are the limitations?’, possible answers: ‘Severely limited’, ‘Moderately limited’) and the duration of the limitations (‘How long have your limitations lasted?’, possible answers: ‘Less than 6 months’, ‘6 months and longer’).

To survey self-rated health, the question ‘How is your health in general?’ was used with the response categories ‘Very good’, ‘Good’, ‘Fair’, ‘Bad’, and ‘Very bad’ [17]. In the analyses, the proportions of participants who rated their health as good and very good were contrasted with participants with self-rated fair to very bad health [10].

To assess the presence of depressive symptoms, the internationally established 8-item Patient Health Questionnaire (PHQ-8) was used [18]. This inquires about the symptoms of major depression in the two weeks before the interview according to DSM-IV (Diagnostic and Statistical Manual of Mental Disorders, 4th edition) [19]. A total of scale values of at least ten out of a maximum of 24 points is considered to indicate the presence of depressive symptoms [20, 21].

The exercise of aerobic physical activities and muscle-strengthening activities was assessed with the German validated version of the European Health Interview Survey – Physical Activity Questionnaires (EHIS-PAQ) [20, 21]. Participants were asked how much time per week they engaged in moderately strenuous aerobic physical activity during leisure time and cycling for locomotion, and how many days per week they engaged in muscle-strengthening activities. On this basis, the proportions of those meeting the World Health Organization (WHO) physical activity recommendations [22] for aerobic physical activities (at least 2.5 hours per week) and muscle-strengthening activities (at least two days per week) were calculated [23].

Fruit consumption was assessed with the question ‘How often do you consume fruit, including freshly squeezed fruit juices?’. Response categories were ‘Daily or several times a day’, ‘4 to 6 times a week’, ‘1 to 3 times a week’, ‘Less than once a week’, and ‘Never’. A similar question was used on vegetable consumption (consumption of vegetables or salad, including freshly squeezed vegetable juices). In each case, the proportion of individuals with daily fruit or vegetable consumption was calculated [24, 25].

Regarding smoking status, the question ‘Do you smoke?’ was asked, with the response categories ‘Yes, daily’, ‘Yes, occasionally’, ‘No, not anymore’, and ‘I have never smoked’. Smokers are those who had answered the question in the affirmative [26].

Alcohol consumption was recorded in GEDA 2014/2015-EHIS using an instrument adapted from the Alcohol Use Disorder Identification Test – Consumption Questions (AUDIT-C) [27]: first the frequency of alcohol consumption in the last twelve months was asked, then, differentiated by weekdays (Monday to Thursday) and weekends (Friday to Sunday), the amount of alcohol consumed based on so-called standard drinks. From this information, the average consumption in grams of pure alcohol per day and the proportion of people exceeding the limits of more than 10g of pure alcohol/day for women and more than 20g of pure alcohol/day for men can be determined. This corresponds to the proportion of those who consume alcohol in risky amounts [28–30].

Outpatient medical utilisation was surveyed with the question ‘When was the last time you consulted a general practitioner or family doctor on your own behalf?’ and a corresponding question for specialist utilisation. The response categories were ‘Less than 12 months ago’, ‘12 months ago or longer’, and ‘Never’. The proportion of those who had used primary care or specialist care at least once in the 12 months prior to the survey was calculated [13]. Use of inpatient care was determined by the question ‘In the past 12 months have you been in hospital as an inpatient, that is overnight or longer?’ [14]. The use of nursing care services was determined by the question ‘In the past 12 months, have you yourself used or received any home care services?’ Both questions could be answered with ‘Yes’ or ‘No’.

### 2.3 Statistical analyses

The analyses are based on data from 23,372 participating persons aged 18 years and older (12,747 women, 10,625 men) with valid information on illness-related permanent limitations as well as officially recognised disabilities. Whether differences in health status, health behaviour, and health care exist between people with and without impairments and disabilities was analysed using selected parameters. Prevalence with 95% confidence intervals and p-values from multivariate log-Poisson regressions were calculated. Regression analyses by sex are controlled for age and socioeconomic status (SES), and regression analyses by sex and age are controlled for SES only. A statistically significant difference between women and men with and without impairments and disabilities is assumed when the p-value is less than 0.05.

Calculations were performed using a weighting factor that corrects for deviations of the sample from the population structure (as of 31.12.2014) in terms of sex, age, type of municipality and education level. Type of municipality reflects the degree of urbanisation and corresponds to the regional distribution in Germany. The International Standard Classification of Education (ISCED) was used to classify the school and vocational degrees of participants [31]. All analyses were conducted using Stata 17.0 survey procedures (Stata Corp., College Station, TX, USA, 2015). A detailed description of the GEDA 2014/2015-EHIS methodology can be found elsewhere [32, 33].

## 3. Results

**Figure 1:**
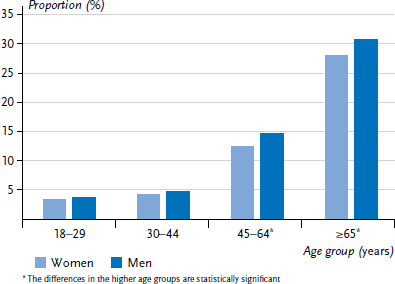
Proportion of women and men with impairments and disabilities by age (n=1,406 women, n=1,505 men) Source: GEDA 2014/2015-EHIS

Of those participating in GEDA 2014/2015-EHIS, 13.5% were affected by impairments and disabilities (women 13.1%, men 13.9%). The proportion of persons with impairments and disabilities increases significantly with age, from 3.4% for women and 3.7% for men aged 18 to 29 years to 27.8% for women and 30.6% for men aged 65 years and older (Figure 1).

###  

#### Self-rated health and depressive symptoms

**Figure 2:**
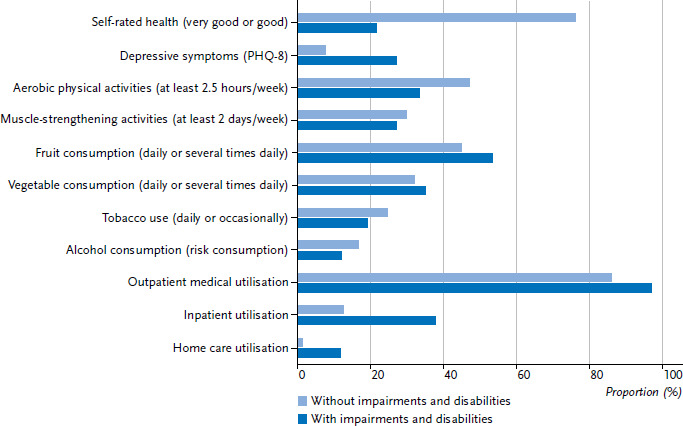
Health status among persons with and without impairments and disabilities (n=2,911 persons with and n=20,461 persons without impairments and disabilities) Source: GEDA 2014/2015-EHIS

Only about one-fifth (21.5%) of persons with impairments and disabilities rate their health as good or very good, in contrast to about three-quarters of persons without impairments and disabilities (76.0%, Figure 2 and Table 1). In this context, women rate their health worse than men on average: 18.8% of women and 24.1% of men with impairments and disabilities report good or very good health (Table 1). The proportion of those who rate their health as very good or good decreases with age; this is true for both women and men with and without impairments and disabilities. Women and men with impairments and disabilities rate their health worse in all age groups (Annex Table 1 and Annex Table 2).

Depressive symptoms in the previous two weeks are present in 31.3% of women and 23.0% of men with impairments and disabilities. Of those without impairments and disabilities, significantly fewer women and men are affected, at 8.6% and 6.3%, respectively (Table 1). With increasing age, the proportion of persons with depressive symptoms decreases; this is evident for both women and men with and without impairments and disabilities. In all age groups, women and men with impairments and disabilities are more frequently affected by depressive symptoms (Annex Table 1 and Annex Table 2).

#### Health behaviour

Women and men with impairments and disabilities are less likely to engage in aerobic physical activities (28.9% and 37.6%, respectively) than women and men without impairments and disabilities (44.7% and 49.6%, respectively). However, the results by age show that this is not equally true for all age groups: for example, among those with impairments and disabilities, it is mainly women in young adulthood (18 to 29 years) and older adulthood (65 years and older) and men in late middle and older adulthood (45 years and older) who engage in aerobic physical activities less often than their peers without impairments and disabilities (Annex Table 1 and Annex Table 2). The picture is different for physical activities for muscle strengthening: Here, there is little difference between the two groups (women 24.4% and 28.1%, men 44.7% and 49.6%; Table 2). Only 65-year-old and older women with impairments and disabilities perform muscle-strengthening exercises less frequently than women of the same age without impairments and disabilities (Annex Table 1).

More than half of women – 59.6% of women with and 53.1% without impairments and disabilities – consume fruit daily, compared with 47.1% and 36.6% of men, respectively (Table 3). There are no significant differences between people with and without impairments and disabilities (Figure 2 and Table 1); this is true for all age groups (Annex Table 1 and Annex Table 2). 41.1% of women with and 40.3% of women without impairments and disabilities consume vegetables daily. For men, this is true for 28.8% and 23.1%, respectively. Differences by age are only observed among women: Women aged 65 years and older with impairments and disabilities are less likely to eat vegetables daily than women of the same age who are not affected by impairments and disabilities (Annex Table 1).

16.0% of women and 22.1% of men with impairments and disabilities reported current smoking, compared with 21.6% and 27.7% of women and men without impairments and disabilities, respectively (Table 4). Results by age show that there are differences in tobacco use between persons with and without impairments and disabilities in some age groups: Women in early middle adulthood (30 to 44 years) with impairments and disabilities were more likely to report current smoking than women of the same age without impairments and disabilities. The same is true for men with and without impairments and disabilities in late middle adulthood (45 to 64 years). In contrast, men in older adulthood (65 years and older) with impairments and disabilities were less likely to report current smoking than men of the same age without impairments and disabilities (Annex Table 1 and Annex Table 2).

In contrast, when it comes to alcohol consumption, people with impairments and disabilities have healthier lifestyles (Figure 2 and Table 1): risky alcohol consumption is present in 8.6% of women and 15.3% of men with impairments and disabilities and in 14.8% of women and 18.6% of men without impairments and disabilities (Table 4). However, the age-stratified results suggest that lower alcohol consumption among people with impairments and disabilities emerges later in adulthood, among women 45 years of age and older and among men 65 years of age and older (Annex Table 1 and Annex Table 2).

#### Utilisation of health care services

At 98.2% and 95.7%, almost all women and men with impairments and disabilities, respectively, use outpatient medical services in the twelve months prior to the survey. Among people without impairments and disabilities, utilisation is lower with 89.9% for women and 82.0% for men. There are also clear differences in the use of hospital treatment. At 38.5% for women and 36.6% for men, utilisation is more than twice as high among people with impairments and disabilities than among people without impairments and disabilities, at 13.3% and 11.8%, respectively (Table 5). Higher use of outpatient medical and inpatient services by people with impairments and disabilities is seen among women in all age groups, and among men only from early middle adulthood (30 years and older) (Annex Table 1 and Annex Table 2).

Home care services are also used much more by persons with impairments and disabilities (Figure 2 and Table 1). Moreover, there is a clear sex difference: 15.0% of women and 8.7% of men with impairments and disabilities use outpatient care, compared with only 2.0% of women and 0.6% of men without impairments and disabilities (Table 5). While higher use of outpatient caregivers is evident for men in all age groups, this is true for women only from early middle adulthood (30 years and older) (Annex Table 1 and Annex Table 2).

## 4. Discussion

People with impairments and disabilities perceive their health as significantly worse than people without impairments and disabilities. They also have poorer mental health, as shown by the higher prevalence of depressive symptoms. This leads to an increased need for medical care and is reflected in a higher utilisation of outpatient, inpatient and home care services. Overall, poorer health is seen in women than in men and with increasing age. The differences in health behaviour are less clear. Persons with impairments and disabilities are less likely to engage in aerobic physical activities than persons without impairments and disabilities; there are almost no differences in muscle-strengthening activities and in fruit and vegetable consumption. Smoking prevalences differ mainly in middle and older age, with partly lower, partly higher values for people with impairments and disabilities. Risky alcohol consumption, on the other hand, is less frequent among persons with impairments and disabilities. In general, women are more health-conscious than men.

###  

#### Self-rated health and depressive symptoms

Significantly poorer health among people with impairments and disabilities can be deduced from many studies [34]. Regarding self-rated health, the GEDA data can be compared with the data of the SOEP, which were analysed for the Third Report on Participation (2021) [3], and of the Representative Survey on the Participation of People with Disabilities (participation survey) [35]. The differences in the concrete figures are mainly related to different survey instruments on subjective health and the different operationalisation of impairments and disabilities (see [3]). According to the SOEP data, 13% of people with impairments and 60% of people without impairments assessed their health as good or very good, i.e. less than in the GEDA study (21.5% and 76.0%). The SOEP analyses also show poorer subjective health among women than among men, but there was no clear increase in poorer health with age [3]. In the participation survey, not officially recognised but self-assessed disabilities were considered. According to the first results, 94% of the non-impaired, 73% of the impaired and 25% of the persons with self-assessed disability in private households rated their health as good or very good [35]. Whether impaired persons are considered self-assessed disabled depends on the severity of the impairment in combination with the severity of the limitation in everyday activities. The participation survey also shows that subjective health was rated differently depending on the type of the most severe impairment: The proportion of those who rated their health as good or very good was highest for people with visual impairment (69%), addiction (64%) and hearing impairment (63%), and lowest for impairment due to pain (41%), emotional or psychological problems (39%) and moving (35%) [35].

Population-wide data on depressive symptoms among people with impairments and disabilities are only available for Germany from the GEDA study, which also serves as the data basis for the Third Report on Participation. High psychological stress among women with impairments and disabilities is shown in the study on life situations of and pressures on disabled women in Germany by the Federal Ministry for Family Affairs, Senior Citizens, Women and Youth [36]. Regional and international studies also show that people with disabilities are more frequently affected by mental health problems [37–39]. Overall, the number of people with mental impairments has increased in Germany [3, 8]. The fact that a high proportion of women with disabilities are affected by mental distress may also be related to discrimination and experiences of violence [9, 36].

The relationship between illness and disability is complex [34]. Many impairments and disabilities result from illnesses; conversely, when people with impairments become ill, they are often affected for longer. Impairments can also strongly influence perceptions of health status and also have an impact on mental health. The fact that people with impairments and disabilities show poorer health also results from the definition of impairments, which comprises ‘damage to body structures and functions’ (these also include mental functions) (Info box) [3]. This also applies to people with chronic diseases, which is reflected in the methodology of the present analyses.

#### Health behaviour

There is data from the SOEP on the sporting activity of people with impairments and disabilities in Germany, which were analysed for the Third Report on Participation. There, too, it is shown that people with impairments and disabilities do less sport overall: 32% state that they actively do sport every week, compared to 48% of those without impairments. There are hardly any differences between women and men, older people do less sport than younger people [3]. These proportions are similar to those for endurance activities (at least 2.5 hours per week) in our analyses, although the difference in the indicators prevents direct comparisons. According to the data from the participation survey, 34% of impaired persons and 50% of persons with self-assessed disability rarely or never engage in sports, in contrast to 30% of non-impaired persons [35]. Reasons for the inactivity of people with impairments and disabilities can be that there are no inclusive offers or that sports facilities are not accessible. But the feeling of not being able to perform certain sporting activities or – in the case of physical impairments – facing health obstacles can also play a role [3, 40]. On the other hand, sporting activity for people with disabilities can increase mobility in everyday life and contribute to physical and mental well-being [41, 42]. The promotion of inclusive sport – both popular and competitive sport – is one of the goals of the Federal Government’s National Action Plan to implement the UN Convention on the Rights of Persons with Disabilities (NAP 2.0) [43].

According to the available analyses, fruit and vegetables are consumed with similar frequency by people with and without impairments and disabilities; so far there is no data comparable to those reported here. In the Report on Participation, the proportion of respondents with an awareness of healthy eating is analysed with SOEP data [44]. Differences become apparent, especially among young men. 35% of 18- to 29-year-old men with impairments and 15% of those of the same age without impairments do not pay attention to health-conscious nutrition; among women and older men, however, the differences were small [3]. As with physical activity, fruit and vegetable consumption has health-promoting effects and may be reduced due to, for example, functional and mobility impairments that may make it difficult to access and prepare these foods [44].

The results reported here on smoking differ from the analyses of the SOEP data from 2018 presented in the Third Report on Participation: at around 23% for women and 30% for men, the prevalence there is seven to eight percentage points higher than in GEDA (16.0% and 22.1%, respectively). Analyses of SOEP data differentiated by age show significantly higher prevalence in people with disabilities up to the age of 65 years, with the prevalences levelling off at older ages. This trend can also be seen in the GEDA data. The differences could be related, for example, to the different operationalisation of impairments and disabilities, but also to differences in the survey methodology. A comparison of smoking prevalences in the general population shows that these are partly higher and partly lower in the RKI data than in the data from the SOEP [45]. Higher prevalence of tobacco consumption among people with impairments and disabilities are also reported in international studies [46, 47].

The fact that people with impairments and disabilities consume alcohol to a lesser extent than people without impairments and disabilities is also described in the Third Report on Participation, which uses the SOEP data from 2018. According to the report, 27% of people with impairments and 33% of people without impairments consumed alcohol on a weekly basis. 32% of people with and 18% of people without impairments and disabilities stated that no alcohol was consumed at all [3]. For the group of people with cognitive disabilities, studies show a lower prevalence of alcohol consumption, but those who do consume alcohol are at higher risk for alcohol abuse [48–51].

#### Health services utilisation

Due to their poorer health status on average, people with impairments and disabilities use health services to a greater extent than people without impairments and disabilities. This is not only evident with regard to the 12-month prevalence of the use of medical services, inpatient care or nursing care, but also when looking at contacts with doctors [3]. The Third Report on Participation states that a large proportion of medical practices are still not accessible [3]. Accessibility does not only mean that ground-level entrances, lifts or wheelchair-accessible practice rooms are available, but also includes, for example, flexible examination furniture, orientation aids for the visually impaired as well as accessible communication and information, for example in sign language or simple language [52–54]. Depending on the type of disability, different barriers play a role. A study on the use of health care by people with cognitive disabilities did not show a general underuse in the outpatient sector, but there was a less frequent use of cancer screening examinations [55]. An analysis of the Swiss Health Survey of 2002 also found that persons with disabilities made more use of services and often use the services more intensively [56]. However, such utilisation data cannot be used to derive any statements on the quality and needs-based nature of care; this would require further – also qualitative – studies. The satisfaction of women with disabilities with their health care is addressed in the study on life situations of and pressures on disabled women in Germany: accordingly, 20% of women with disabilities living in households were rather dissatisfied to very dissatisfied with their health care [36]. The Participation Survey also showed gaps in care: people with self-assessed disabilities more often reported not having access to necessary counselling and treatment than people with and without impairments. This was most frequently the case for psychological or psychiatric counselling and treatment (8.9%), rehabilitation (7.2%) and psychiatric facilities (6.2%). Overall, 21.4% of people with self-assessed disabilities reported not having access to necessary counselling or treatment from at least one agency, and this was particularly common among women and among people with a migration background [3].

#### Strengths and limitations

GEDA 2014/2015-EHIS is a population-representative survey with a large number of participants. However, the method also has limitations that are particularly relevant for people with impairments and disabilities. Participation in surveys aimed at the general population can be difficult for people with disabilities, for example, if people with visual impairments cannot fill out the paper or online questionnaires used for the survey, or can do so only with difficulty. This can result in under-representation and bias in the results due to selective non-participation (selection bias) [57]. Also, people who do not live in their own households or with their families, but in residential facilities or nursing homes, were not included in the survey. Furthermore, there are special limitations for individual indicators. For example, self-reported use of health care services can be associated with recall bias [58]; however, this applies more to the number of contacts than to whether doctors in private practice were used at all. Recall bias is also more likely if a longer period than the last twelve months is recorded [59]. Another limitation is the socially desirable response behaviour, which plays a role especially for indicators such as tobacco and alcohol consumption [26, 30]. Another limitation is the age of the data source (2014/2015). However, there is no recent data available in RKI health monitoring in which the presence of impairments and disabilities can be analysed in combination; the analyses showed that 2.6% of people without an officially recognised disability reported a severe and permanent illness-related limitation.

#### Conclusion and outlook

Like other reports and studies, our results show the health inequality between people with and without impairments and disabilities. Women with impairments and disabilities are (partly) more affected by health disadvantages than men. The Third Report on Participation points out that health and participation are closely linked and that special attention must be paid to persons with multiple disadvantages in the sense of intersecting forms of discrimination (intersectionality) [3]. Overall, the results presented here are only an initial overview. In order to be able to make more detailed statements on individual groups of people with impairments, for example according to age, social situation and migration history, further and up-to-date data and analyses are necessary – also in view of the fact that people with impairments and disabilities are a very heterogeneous group, in different life situations and with different needs. This heterogeneity is taken into account, for example, in the participation survey, which was also designed in a participatory manner and provided for the involvement of the respondents [60]. It would be desirable to conduct such a survey also on health topics or to supplement health surveys with corresponding questions in order to obtain reliable data on the health situation of people with impairments and disabilities, also as a basis for (health) policy decisions. For example, important findings were obtained from the study on the living conditions of women with disabilities and impairments, for example on psychological stress, satisfaction with one’s own health and health care or the use of medication [36]. There is a particular need for research in the field of prevention and health promotion [3, 61]. For less specific questions, data is also available in many epidemiological studies that could be analysed with regard to impairments and disabilities [5]. The UNCRPD requires partner states to collect data in order to develop and implement policies to implement the Convention (Article 31) [4].

Comparing our findings with the UNCRPD, further needs for action arise from a public health perspective. Article 25 states that persons with disabilities shall be provided with ‘the same range, quality and standard of free or affordable health care and programmes as provided to other persons’ and ‘health services needed by persons with disabilities specifically because of their disabilities’ [4]. Equal participation in sports activities (Art. 30) is also contained in the UNCRPD [4]. It follows from this that – in addition to care aspects such as the accessibility of medical practices, more therapy offers for people with mental disorders and the adaptation of inpatient care to the needs of people with impairments and disabilities – targeted prevention and health promotion continue to be important goals. In the course of demographic change, the number of people with impairments and disabilities has increased in recent decades and this development will continue in the future [3, 8]. Therefore, the aspects mentioned are also important for future health care planning.

## Key statements

- A fifth of the persons with impairments and disabilities rate their health as good or very good, in contrast to three quarters of the persons without impairments and disabilities.
- Depressive symptoms exist in about 30% of women and 23% of men with impairments and disabilities, but in about 9% of women and 6% of men without impairments and disabilities.
- In some areas of health behaviour, people with and without impairments and disabilities differ, for example, the former do less aerobic physical activities and consume less alcohol in risky amounts.
- Within one year, more than 95% of persons with impairments and disabilities make use of outpatient medical services, a higher utilisation than in persons without impairments and disabilities.
- In order to describe the health situation of people with impairments and disabilities and to determine the need for action in health policy, further data is essential.

## Figures and Tables

**Table 1:** Self-rated health and depressive symptoms among women and men with and without impairments and disabilities (n=1,406 women with/n=11,341 without impairments and disabilities, n=1,505 men with/n=9,120 without impairments and disabilities) Source: GEDA 2014/2015-EHIS

	Self-rated health (very good or good)			Depressive symptoms (PHQ-8)		
	%	(95% CI)	p-value*	%	(95% CI)	p-value*
**Women**						
With impairments and disabilities	18.8	(16.3–21.5)	<0.001	31.3	(28.4–34.4)	<0.001
Without impairments and disabilities	74.5	(73.4–75.6)	Ref.	8.6	(8.0–9.3)	Ref.
**Men**						
With impairments and disabilities	24.1	(21.6–26.8)	<0.001	23.0	(20.3–25.9)	<0.001
Without impairments and disabilities	77.6	(76.5–78.7)	Ref.	6.3	(5.7–7.1)	Ref.
**Total**						
With impairments and disabilities	21.5	(19.6–23.4)	<0.001	27.1	(25.1–29.1)	<0.001
Without impairments and disabilities	76.0	(75.2–76.8)	Ref.	7.5	(7.0–8.0)	Ref.

CI = confidence interval, Ref. = reference group, PHQ-8 = 8-item Patient Health Questionnaire

^*^ p-value from multivariate log-Poisson regressions

**Table 2:** Aerobic physical activities and muscle-strengthening activities among women and men with and without impairments and disabilities (n=1,406 women with/n=11,341 without impairments and disabilities, n=1,505 men with/n=9,120 without impairments and disabilities) Source: GEDA 2014/2015-EHIS

	Aerobic physical activities (at least 2.5 hours/week)			Muscle-strengthening activities (at least 2 days/week)		
	%	(95% CI)	p-value*	%	(95% CI)	p-value*
**Women**						
With impairments and disabilities	28.9	(25.9–32.0)	<0.001	24.4	(21.6–27.5)	0.539
Without impairments and disabilities	44.7	(43.3–46.1)	Ref.	28.1	(27.1–29.1)	Ref.
**Men**						
With impairments and disabilities	37.6	(34.2–41.2)	<0.001	29.6	(26.6–32.8)	0.812
Without impairments and disabilities	49.6	(48.8–51.9)	Ref.	31.5	(30.4–32.7)	Ref.
**Total**						
With impairments and disabilities	33.3	(31.1–35.7)	<0.001	27.0	(24.9–29.3)	0.687
Without impairments and disabilities	47.1	(46.0–48.3)	Ref.	29.8	(28.9–30.6)	Ref.

CI = confidence interval, Ref. = reference group

^*^ p-value from multivariate log-Poisson regressions

**Table 3:** Fruit and vegetable consumption among women and men with and without impairments and disabilities (n=1,406 women with/n=11,341 without impairments and disabilities, n=1,505 men with/n=9,120 without impairments and disabilities) Source: GEDA 2014/2015-EHIS

	Fruit consumption (daily or several times daily)			Vegetable consumption (daily or several times daily)		
	%	(95% CI)	p-value*	%	(95% CI)	p-value*
**Women**						
With impairments and disabilities	59.6	(56.2–63.0)	0.069	41.4	(38.7–44.2)	0.145
Without impairments and disabilities	53.1	(51.9–54.4)	Ref.	40.3	(39.1–41.6)	Ref.
**Men**						
With impairments and disabilities	47.1	(43.9–50.4)	0.394	28.8	(26.0–31.8)	0.516
Without impairments and disabilities	36.6	(35.3–37.9)	Ref.	23.1	(21.9–24.2)	Ref.
**Total**						
With impairments and disabilities	53.3	(50.9–55.6)	0.083	35.0	(33.1–37.0)	0.610
Without impairments and disabilities	45.0	(44.0–46.1)	Ref.	31.9	(30.9–32.8)	Ref.

CI = confidence interval, Ref. = reference group

^*^ p-value from multivariate log-Poisson regressions

**Table 4:** Tobacco and alcohol consumption among women and men with and without impairments and disabilities (n=1,406 women with/n=11,341 without impairments and disabilities, n=1,505 men with/n=9,120 without impairments and disabilities) Source: GEDA 2014/2015-EHIS

	Tobacco use (daily or occasionally)			Alcohol consumption (risky consumption)		
	%	(95% CI)	p-value*	%	(95% CI)	p-value*
**Women**						
With impairments and disabilities	16.0	(13.7–18.6)	0.108	8.6	(7.0–10.6)	<0.001
Without impairments and disabilities	21.6	(20.6–22.6)	Ref.	14.8	(13.9–15.8)	Ref.
**Men**						
With impairments and disabilities	22.1	(19.6–24.7)	0.278	15.3	(13.2–17.6)	0.005
Without impairments and disabilities	27.7	(26.5–28.9)	Ref.	18.6	(17.6–19.6)	Ref.
**Total**						
With impairments and disabilities	19.1	(17.5–20.8)	0.034	12.0	(10.5–13.6)	<0.001
Without impairments and disabilities	24.6	(23.8–25.4)	Ref.	16.7	(16.0–17.4)	Ref.

CI = confidence interval, Ref. = reference group

^*^ p-value from multivariate log-Poisson regressions

**Table 5:** Utilisation of outpatient medical services, inpatient services, and home care services among women and men with and without impairments and disabilities (n=1,406 women with/n=11,341 without impairments and disabilities, n=1,505 men with/n=9,120 without impairments and disabilities) Source: GEDA 2014/2015-EHIS

	Outpatient medical utilisation			Inpatient utilisation			Utilisation of home care services		
	%	(95% CI)	p-value*	%	(95% CI)	p-value*	%	(95% CI)	p-value*
**Women**									
With impairments and disabilities	98.2	(97.2–98.9)	<0.001	38.5	(35.6–41.4)	<0.001	15.0	(12.8–17.6)	<0.001
Without impairments and disabilities	89.9	(89.1–90.7)	Ref.	13.3	(12.5–14.1)	Ref.	2.0	(1.8–2.4)	Ref.
**Men**									
With impairments and disabilities	95.7	(94.2–96.9)	<0.001	36.9	(34.1–39.8)	<0.001	8.7	(7.1–10.6)	<0.001
Without impairments and disabilities	82.0	(81.0–83.1)	Ref.	11.8	(11.0–12.7)	Ref.	0.6	(0.4–0.8)	Ref.
**Total**									
With impairments and disabilities	97.0	(96.1–97.7)	<0.001	37.7	(35.6–39.8)	<0.001	11.8	(10.4–13.4)	<0.001
Without impairments and disabilities	86.1	(85.3–86.7)	Ref.	12.6	(12.0–13.1)	Ref.	1.3	(1.1–1.5)	Ref.

CI = confidence interval, Ref. = reference group

^*^ p-value from multivariate log-Poisson regressions

## Appendix

**Annex Table 1:** Health situation among women with and without impairments and disabilities by age (n=1,406 women with and n=11,341 without impairments and disabilities) Source: GEDA 2014/2015-EHIS

	18–29 years				30–44 years			
	With ID		Without ID		With ID		Without ID	
**Self-rated health (very good or good)**								
% (95% Cl)	20.5	(11.2–34.6)	82.9	(80.7–84.9)	23.2	(15.5–33.2)	83.2	(81.3–84.9)
p-value*	<0.001		Ref.		<0.001		Ref.	
**Depressive symptoms (PHQ-8)**								
% (95% Cl)	55.5	(39.8–70.0)	15.0	(13.2–17.1)	42.9	(32.3–54.1)	9.1	(7.8–10.5)
p-value*	<0.001		Ref.		<0.001		Ref.	
**Aerobic physical activities (at least 2.5 hours per week)**								
% (95% Cl)	23.9	(13.4–39.1)	46.1	(43.0–49.1)	32.7	(23.6–43.3)	39.3	(37.1–41.6)
p-value*	0.003		Ref.		0.358		Ref.	
**Muscle-strengthening activities (at least 2 days per week)**								
% (95% Cl)	28.3	(17.2–43.0)	34.6	(32.1–37.2)	24.5	(16.1–35.5)	21.2	(19.5–23.0)
p-value*	0.640		Ref.		0.351		Ref.	
**Fruit consumption (daily or several times daily)**								
% (95% Cl)	33.6	(21.1–49.0)	38.3	(35.7–41.1)	46.4	(35.9–57.2)	44.5	(42.2–46.9)
p-value*	0.758		Ref.		0.314		Ref.	
**Vegetable consumption (daily or several times daily)**								
% (95% Cl)	33.1	(21.1–47.8)	31.9	(29.5–34.3)	35.8	(25.8–47.3)	38.6	(36.2–41.1)
p-value*	0.668		Ref.		0.942		Ref.	
**Tobacco use (daily or occasionally)**								
% (95% Cl)	17.8	(8.6–33.3)	28.4	(26.1–30.8)	40.5	(29.4–52.6)	26.2	(24.1–28.4)
p-value*	0.112		Ref.		0.047		Ref.	
**Alcohol consumption (risky consumption)**								
% (95% Cl)	4.6	(1.6–12.6)	13.2	(11.5–15.2)	10.9	(5.7–19.8)	11.1	(9.5–12.9)
p-value*	0.063		Ref.		0.764		Ref.	
**Outpatient medical utilisation**								
% (95% Cl)	100.0	100.0	90.1	(88.3–91.6)	99.6	(97.5–99.9)	87.4	(85.7–88.9)
p-value*	<0.001		Ref.		<0.001		Ref.	
**Inpatient utilisation**								
% (95% Cl)	34.0	(21.6–49.1)	14.3	(12.3–16.6)	32.5	(23.2–43.3)	10.3	(9.0–11.9)
p-value*	0.004		Ref.		<0.001		Ref.	
**Home care utilisation**								
% (95% Cl)	2.6	(0.4–13.9)	2.3	(1.6–3.4)	7.8	(3.9–14.9)	3.5	(2.8–4.2)
p-value^*^	0.926		Ref.		0.023		Ref.	
	**45–64 years**				**≥65 years**			
	**With ID**		**Without ID**		**With ID**		**Without ID**	
**Self-rated health (very good or good)**								
% (95% Cl)	21.9	(17.8–26.6)	74.5	(72.9–76.1)	16.1	(12.9–19.8)	57.5	(54.4–60.5)
p-value*	<0.001		Ref.		<0.001		Ref.	
**Depressive symptoms (PHQ-8)**								
% (95% Cl)	36.1	(31.4–41.0)	8.3	(7.3–9.4)	24.7	(21.0–28.8)	3.1	(2.2–4.2)
p-value*	<0.001		Ref.		<0.001		Ref.	
**Aerobic physical activities (at least 2.5 hours per week)**								
% (95% Cl)	41.0	(35.9–46.3)	49.0	(47.2–50.9)	20.4	(16.7–24.7)	42.2	(39.1–45.5)
p-value*	0.075		Ref.		<0.001		Ref.	
**Muscle-strengthening activities (at least 2 days per week)**								
% (95% Cl)	30.0	(25.4–35.1)	29.2	(27.6–30.8)	20.3	(16.7–24.3)	28.4	(26.1–30.7)
p-value*	0.311		Ref.		0.003		Ref.	
**Fruit consumption (daily or several times daily)**								
% (95% Cl)	51.3	(46.5–56.1)	53.8	(51.9–55.7)	68.6	(63.6–73.2)	74.6	(72.1–76.9)
p-value*	0.661		Ref.		0.057		Ref.	
**Vegetable consumption (daily or several times daily)**								
% (95% Cl)	40.8	(36.2–45.6)	39.6	(37.8–41.5)	43.2	(39.2–47.3)	50.6	(47.8–53.5)
p-value*	0.139		Ref.		0.009		Ref.	
**Tobacco use (daily or occasionally)**								
% (95% Cl)	26.1	(21.6–31.1)	23.7	(22.3–25.2)	6.2	(4.4–8.5)	6.8	(5.7–8.1)
p-value*	0.856		Ref.		0.892		Ref.	
**Alcohol consumption (risky consumption)**								
% (95% Cl)	9.2	(6.8–12.4)	18.6	(17.1–20.1)	8.3	(5.9–11.4)	14.2	(12.4–16.3)
p-value*	<0.001		Ref.		0.006		Ref.	
**Outpatient medical utilisation**								
% (95% Cl)	97.5	(95.2–98.7)	89.8	(88.6–90.8)	98.4	(96.8–99.2)	92.9	(91.3–94.2)
p-value*	<0.001		Ref.		<0.001		Ref.	
**Inpatient utilisation**								
% (95% Cl)	35.9	(31.3–40.8)	11.0	(9.9–12.2)	41.2	(37.1–45.4)	19.7	(17.4–22.2)
p-value*	<0.001		Ref.		<0.001		Ref.	
**Home care utilisation**								
% (95% Cl)	6.2	(4.2–8.9)	0.5	(0.3–0.9)	22.6	(19.0–26.5)	2.8	(2.0–3.8)
p-value^*^	<0.001		Ref.		<0.001		Ref.	

With ID = with impairments and disabilities, without ID = without impairments and disabilities, CI = Confidence interval, Ref. = Reference group, PHQ-8 = 8-Item Patient Health Questionnaire

^*^ p-value from multivariate log-Poisson regressions

**Annex Table 2:** Health status among men with and without impairments and disabilities (n=1,505 men with and n=9,120 persons without impairments and disabilities) Source: GEDA 2014/2015-EHIS

	18–29 years				30–44 years			
	With ID		Without ID		With ID		Without ID	
**Self-rated health (very good or good)**								
% (95% Cl)	46.0	(30.1–62.7)	91.2	(89.2–92.8)	33.8	(23.5–46.0)	83.6	(81.4–85.5)
p-value*	<0.001		Ref.		<0.001		Ref.	
**Depressive symptoms (PHQ-8)**								
% (95% Cl)	34.2	(19.8–52.2)	8.6	(6.8–10.7)	40.1	(28.4–53.1)	7.8	(6.4–9.5)
p-value*	<0.001		Ref.		<0.001		Ref.	
**Aerobic physical activities (at least 2.5 hours per week)**								
% (95% Cl)	44.1	(28.0–61.6)	57.0	(53.8–60.1)	32.4	(21.2–46.0)	54.5	(51.7–57.2)
p-value*	0.270		Ref.		0.121		Ref.	
**Muscle-strengthening activities (at least 2 days per week)**								
% (95% Cl)	36.1	(21.4–53.8)	43.9	(41.1–46.8)	23.5	(14.5–35.9)	28.9	(26.7–31.3)
p-value*	0.422		Ref.		0.613		Ref.	
**Fruit consumption (daily or several times daily)**								
% (95% Cl)	36.0	(21.6–53.5)	25.2	(22.5–28.1)	33.0	(22.3–45.9)	28.2	(25.9–30.6)
p-value*	0.139		Ref.		0.296		Ref.	
**Vegetable consumption (daily or several times daily)**								
% (95% Cl)	29.1	(17.0–45.1)	19.7	(17.4–22.2)	19.8	(12.1–30.6)	19.1	(17.1–21.2)
p-value*	0.172		Ref.		0.803		Ref.	
**Tobacco use (daily or occasionally)**								
% (95% Cl)	20.7	(10.5–36.6)	35.1	(32.0–38.4)	46.7	(35.8–57.9)	34.9	(32.4–37.6)
p-value*	0.065		Ref.		0.204		Ref.	
**Alcohol consumption (risky consumption)**								
% (95% Cl)	9.3	(3.2–24.2)	17.6	(15.5–19.9)	11.4	(5.7–21.3)	13.7	(12.0–15.7)
p-value*	0.232		Ref.		0.485		Ref.	
**Outpatient medical utilisation**								
% (95% Cl)	83.8	(66.7–93.0)	77.9	(75.2–80.4)	92.8	(83.6–97.0)	77.0	(74.6–79.3)
p-value*	0.330		Ref.		<0.001		Ref.	
**Inpatient utilisation**								
% (95% Cl)	17.5	(8.0–34.2)	8.2	(6.6–10.2)	37.3	(26.1–50.0)	8.2	(6.8–9.8)
p-value*	0.078		Ref.		<0.001		Ref.	
**Home care utilisation**								
% (95% Cl)	26.5	(13.6–45.2)	0.1	(0.0–0.4)	11.0	(4.8–23.1)	0.4	(0.2–1.0)
p-value^*^	<0.001		Ref.		<0.001		Ref.	
	**45–64 years**				**≥65 years**			
	**With ID**		**Without ID**		**With ID**		**Without ID**	
**Self-rated health (very good or good)**								
% (95% Cl)	23.1	(19.4–27.1)	73.3	(71.4–75.1)	21.1	(17.9–24.9)	62.3	(59.4–65.1)
p-value*	<0.001		Ref.		<0.001		Ref.	
**Depressive symptoms (PHQ-8)**								
% (95% Cl)	28.7	(24.0–34.0)	6.3	(5.4–7.3)	14.3	(11.4–17.6)	1.7	(1.2–2.5)
p-value*	<0.001		Ref.		<0.001		Ref.	
**Aerobic physical activities (at least 2.5 hours per week)**								
% (95% Cl)	37.0	(32.0–42.3)	47.2	(45.1–49.3)	38.4	(34.3–42.7)	52.5	(49.5–55.5)
p-value*	0.017		Ref.		<0.001		Ref.	
**Muscle-strengthening activities (at least 2 days per week)**								
% (95% Cl)	28.8	(24.2–33.9)	26.0	(24.4–27.7)	30.7	(27.0–34.6)	32.8	(30.4–35.4)
p-value*	0.070		Ref.		0.557		Ref.	
**Fruit consumption (daily or several times daily)**								
% (95% Cl)	32.5	(27.9–37.3)	37.0	(34.9–39.3)	62.8	(58.5–66.8)	60.9	(58.3–63.5)
p-value*	0.105		Ref.		0.388		Ref.	
**Vegetable consumption (daily or several times daily)**								
% (95% Cl)	19.5	(15.9–23.7)	21.9	(20.4–23.6)	37.9	(33.7–42.2)	35.2	(32.4–38.0)
p-value*	0.730		Ref.		0.301		Ref.	
**Tobacco use (daily or occasionally)**								
% (95% Cl)	35.1	(30.5–40.1)	26.9	(25.2–28.7)	7.4	(5.7–9.7)	10.1	(8.6–11.7)
p-value*	0.041		Ref.		0.030		Ref.	
**Alcohol consumption (risky consumption)**								
% (95% Cl)	18.4	(14.8–22.6)	22.1	(20.5–23.9)	13.9	(11.3–17.1)	19.3	(17.3–21.5)
p-value*	0.143		Ref.		0.048		Ref.	
**Outpatient medical utilisation**								
% (95% Cl)	96.3	(93.5–98.0)	83.1	(81.5–84.6)	96.9	(95.1–98.1)	91.9	(90.2–93.2)
p-value*	<0.001		Ref.		<0.001		Ref.	
**Inpatient utilisation**								
% (95% Cl)	37.5	(32.9–42.3)	12.1	(10.9–13.5)	38.3	(34.1–42.7)	20.4	(18.2–22.8)
p-value*	<0.001		Ref.		<0.001		Ref.	
**Home care utilisation**								
% (95% Cl)	3.8	(2.3–6.2)	0.4	(0.2–0.8)	10.5	(8.3–13.2)	1.6	(1.0–2.7)
p-value^*^	<0.001		Ref.		<0.001		Ref.	

With ID = with impairments and disabilities, without ID = without impairments and disabilities, CI = Confidence interval, Ref. = Reference group, PHQ-8 = 8-Item Patient Health Questionnaire

^*^ p-value from multivariate log-Poisson regressions
